# Rare Head and Neck Cancers and Pathological Diagnosis Challenges: A Comprehensive Literature Review

**DOI:** 10.3390/diagnostics14212365

**Published:** 2024-10-23

**Authors:** Daria Maria Filippini, Francesca Carosi, Giulia Querzoli, Matteo Fermi, Ilaria Ricciotti, Gabriele Molteni, Livio Presutti, Maria Pia Foschini, Laura Deborah Locati

**Affiliations:** 1 Division of Medical Oncology, IRCCS Azienda Ospedaliero-Universitaria Sant’Orsola Malpighi, 40138 Bologna, Italy; ilaria.ricciotti@studio.unibo.it; 2 Department of Medical and Surgical Sciences (DIMEC), Alma Mater Studiorum, Università di Bologna, 40138 Bologna, Italy; francesca.carosi3@studio.unibo.it; 3 Pathology Unit, IRCCS Azienda Ospedaliero Universitaria di Bologna, 40138 Bologna, Italy; 4 Department of Otorhinolaryngology—Head and Neck Surgery, IRCCS Azienda Ospedaliero-Universitaria di Bologna, 40138 Bologna, Italy; matteo.fermi3@unibo.it (M.F.); g.molteni@unibo.it (G.M.); livio.presutti@unibo.it (L.P.); 5 Unit of Anatomic Pathology, Department of Biomedical and Neuromotor Sciences (DIBINEM), University of Bologna, Bellaria Hospital, 40139 Bologna, Italy; mariapia.foschini@unibo.it; 6 Department of Internal Medicine and Therapeutics, University of Pavia, 27100 Pavia, Italy; lauradeborah.locati@icsmaugeri.it; 7 Medical Oncology Unit, Istituti Clinici Scientifici Maugeri IRCCS, 27100 Pavia, Italy

**Keywords:** head and neck cancer, rare cancers, histopathological diagnosis, molecular diagnostics, multidisciplinary care

## Abstract

Head and neck cancers (HNCs) arise from anatomically adjacent sites and subsites, with varying etiological factors, diagnostic strategies, prognoses, and treatment approaches. While conventional squamous cell carcinoma (SCC) is the most common histology in the head and neck district, HNCs encompass a variety of rare histopathological entities, categorized into epithelial tumors such as salivary gland cancers, sinonasal tumors, neuroendocrine tumors, malignant odontogenic tumors, and SCC variants versus non-epithelial tumors including soft tissue sarcomas, mucosal melanomas, and hematological malignancies. Rare HNCs (R-HNCs) represent a diagnostic and clinical challenge, requiring histopathological expertise, the availability of peculiar molecular analysis, and the personalization of local and systemic treatments, all guided by a multidisciplinary tumor board. Here, we provide a comprehensive literature review on R-HNCs, emphasizing key histopathological and molecular characteristics that are crucial for guiding treatment decisions. An insight about the latest developments in systemic treatments is also reported.

## 1. Introduction

Head and neck cancers (HNCs) are the seventh most common cancers globally, accounting for more than 660,000 new cases and 325,000 deaths per year [1]. HNCs may arise from mucosal membranes, skin, salivary glands, soft tissues, and craniofacial bones. The majority, nearly 90%, are squamous cell carcinomas (SCCs), with alcohol consumption and smoking habits being the main risk factors [2]. Viral infections such as Epstein–Barr Virus (EBV) or Human Papillomavirus (HPV) represent well-known risk factors for nasopharyngeal cancer (NPC) [3] and for oropharyngeal cancer [4], respectively.

According to the newly developed definition for rare tumors by RARECARE (incidence <6 per 100.000) [5] and to the definition for rare diseases by the European Commission (prevalence <50 per 100.000), HNCs can be defined as rare tumors in Europe according to the incidence of each subsite [6].

Among HNCs, those of the oral cavity had the highest annual crude incidence rate at 48 per million, followed by cancers of the oropharynx and major salivary glands or salivary-gland-type tumors, with incidence rates of 28 and 13 per million, respectively. The incidence rates of epithelial tumors in the nasal cavities, nasopharynx, eye and adnexa, and middle ears were all below five per million [5].

Novel types and subtypes of HNCs have been identified based on molecular patterns, immunohistochemistry (IHC), and unique clinical presentations and outcomes. Many of these entities have been formally recognized as distinct diseases in the latest classifications by the World Health Organization (WHO) and the American Joint Committee on Cancer (AJCC), published in 2022 [7]. Concerning the histopathological features, rare HNCs (r-HNCs) can be divided into epithelial tumors, i.e., salivary glands cancers (SGCs), sinonasal tumors, variants of conventional squamous cell carcinoma (SCC) including nasopharyngeal carcinoma (NPC), neuroendocrine neoplasms (NENs), malignant odontogenic tumors, and ear tumors, and rare non-epithelial tumors, i.e., soft tissue sarcomas, mucosal melanomas, lymphomas, and other hematological malignancies, tumors of uncertain or undetermined malignancy.

R-HNCs are particularly challenging to diagnose due to their low incidence and the overlap in histological features with more common subtypes. The rarity of these tumors often leads to limited clinical experience, making it difficult for pathologists and oncologists to recognize and classify them accurately. Additionally, many r-HNCs exhibit complex and diverse molecular profiles, further complicating their diagnosis [8]. Misdiagnosis or delayed diagnosis can occur, impacting treatment decisions and patient outcomes.

The current diagnostic methods for r-HNCs include a combination of histopathological examination, IHC, and increasingly, molecular analysis. Hematoxylin and eosin (H&E) staining remains the cornerstone for initial tumor classification, but it is often supplemented with IHC to identify the expression of specific protein markers. Molecular techniques, such as next-generation sequencing (NGS), are becoming more widely used to detect gene mutations, fusions, and other alterations that are characteristic of certain rare cancers [i.e., the CRTC1/MAML2 fusion transcript, which arises from the CRTC1/MAML2 translocation, is a major oncogenic driver for mucoepidermoid carcinoma (MEC)]. These methods provide higher sensitivity and specificity, aiding in more precise diagnosis.

Additionally, for HNCs and especially for rare histologies, a multidisciplinary team is required for the elaboration of the appropriate therapeutic strategy, with the contribution of different specialists (surgeons, oncologists, radiologists, and particularly pathologists with expertise in the field of r-HNC).

Here, we provide a review of the current literature about r-HNCs, highlighting the main histopathological as well as molecular features (Table 1), with the aim of describing the complexity and challenges of the diagnosis of these tumors.

## 2. Rare Head and Neck Cancers

### 2.1. Nasopharyngeal Carcinoma (NPC)

The nasopharynx is the narrow tubular passage behind the nasal cavity. Because the nasopharynx is in close proximity to various anatomical structures, tumors arising in adjacent sites can also present clinically as a nasopharyngeal mass.

The most common tumor of the nasopharynx is NPC, a malignant mucosal epithelial neoplasm usually with squamous differentiation [9]. The etiological factors for NPC include genetic, viral, and environmental elements. Men are usually affected more than women (male/female ratio is 2.75). The age distribution differs in endemic areas compared with low-incidence areas. In the latter, the incidence of NPC is higher in adolescents and young adults with a second peak after the age of 65, whereas in endemic areas, the incidence increases after the age of 30, peaks at 40–59 years, and subsequently decreases [10].

Most patients with NPC can present initially with lymph node metastases, often leading to a diagnosis through fine-needle aspiration biopsy [11]. This scenario is common, as NPC frequently metastasizes to cervical lymph nodes before the primary tumor is identified. To raise suspicion of a nasopharyngeal origin, specific diagnostic tools such as in situ hybridization (ISH) for EBV should be employed. On the other hand, immunostaining for LMP1 is not a sensitive method to demonstrate EBV presence. NPC is strongly positive for p63, pancytokeratin, and high-molecular-weight cytokeratins and EMA; CK7 and CK20 are negative [12].

In NPC, three histological subtypes are described: non-keratinizing SCC (NK-NPC) (subdivided into differentiated and undifferentiated, without clinical or prognostic value), keratinizing SCC (K-NPC), and basaloid SCC.

The tumor exhibits a variety of architectural patterns, often within the same tumor, including solid sheets, irregular islands, trabeculae, and isolated discohesive cells, all intermixed with varying numbers of lymphocytes and plasma cells. The density of lymphocytes and plasma cells within the tumor is highly variable. When these inflammatory cells are abundant, they break up the nests and sheets of tumor cells into tiny clusters or single cells, making it difficult to recognize the epithelial nature of the neoplasm [9].

**Figure 1:**
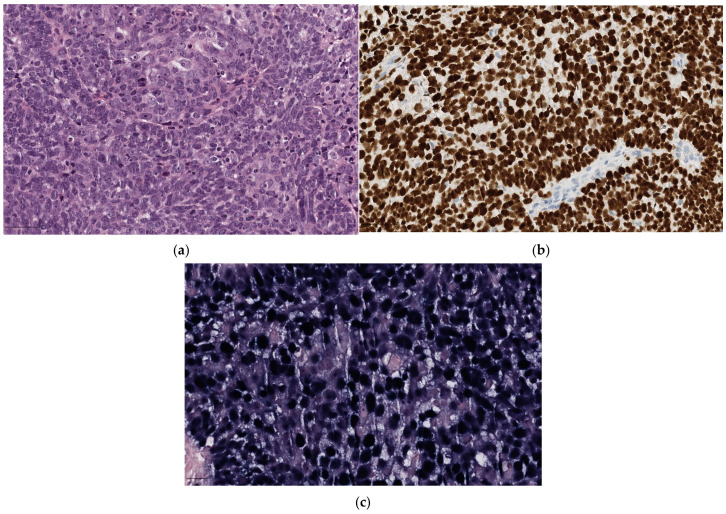
H&E undifferentiated NK-NPC. (**a**) Spindly tumor cells (40×): the nuclei have fine chromatin and small, distinct nucleoli; (**b**) the tumor cells show immunoreactivity for cytokeratin and p63 supporting their epithelial nature; (**c**) positive labeling of the tumor cell nuclei for EBV-encoded small RNAs (EBERs) on in situ hybridization supports the diagnosis of nasopharyngeal carcinoma.

The undifferentiated subtype consists of large neoplastic cells with a syncytial appearance, featuring round to oval vesicular nuclei and large central nucleoli (Figure 1). ISH-EBV (EBER) is positive in nearly all cases of NK-NPC, which is the most common histologic subtype, but shows variable results in K-NPC (more frequent in non-endemic than endemic areas [13]), not always correlated with EBV infection and basaloid SCC, especially in high-incidence ethnic groups.

A wide spectrum of lesions may be considered in the differential diagnosis of NPC, ranging from benign lymphoepithelial lesions to undifferentiated carcinomas, depending on the anatomic region involved. Furthermore, few diffuse large B-cell lymphomas (DLBCLs) are EBV-associated [14]. Immunohistochemical markers demonstrating the epithelial nature with squamous differentiation of the epithelial cells can assist in reaching the correct diagnosis.

Currently, there is no consensus regarding the prognostic significance of the histologic subtype of NPC or of the tumors’ EBV/EBV-encoded RNA (EBER) status, but the detection of circulating EBV-DNA is emerging as a relevant prognostic factor, especially for EBV/EBER NPC: the specific cut-off values of circulating EBV-DNA are related to both the disease stage and cancer-related risk, with lower levels associated with a low stage and better prognosis [15,16].

Due to the role of viral infection in carcinogenesis for NPC, several EBV-specific vaccines have been developed and are currently being evaluated in clinical trials, with both prophylactic and therapeutic intent, using cell-derived virus and peptide platforms [17].

Whereas radiotherapy (RT) remains the mainstay of curative-intent treatment of early-stage NPC, for the recurrent/metastatic setting, the most recent guidelines recommend first-line therapy with chemotherapy (cisplatin and gemcitabine) combined with immunotherapy, based on two randomized phase III clinical trials conducted in Asian patients which showed an improved PFS with the addition of anti-PD-L1 antibodies camrelizumab [18] or toripalimab [19], respectively, even used as maintenance therapy in case of response. Currently, only toripalimab has been approved by the EMA for the first-line treatment of recurrent/metastatic NPCs in combination with standard chemotherapy with cisplatin and gemcitabine.

### 2.2. Salivary Gland Cancers

The salivary glands, both major and minor, are affected by an extraordinary variety of neoplasms. In recent years, the classification of SGCs has experienced significant and transformative changes. Compared to tumors in other organs or systems, SGCs exhibit some of the most extensive morphological, phenotypic, and genotypic diversity found in any single-organ system [20,21].

Major SGCs represent approximately 3–5% of all HNCs and 1% of all tumors [5]. In Europe, their incidence is around 1.39%, with a frequency of approximately 0.67 cases per 100.000 people per year, while minor SGCs are even less frequent. Males, primarily in their sixth and seventh decades of life, are more commonly affected, except for acinic cell carcinoma and adenoid cystic carcinoma (ACC), which tend to occur at a younger age [22].

The etiology of these tumors remains uncertain: the identified risk factors include prior exposure to RT (i.e., MEC) [23,24], viral infections such as EBV, human cytomegalovirus (CMV), and human immunodeficiency virus (HIV), exposure to ultraviolet radiation, interaction with industrial chemicals including nickel [25], and genetic factors [26].

SGCs encompass more than twenty malignant histological subtypes. The morphological variability reflects a complex biological landscape; consequently, accurate diagnosis could be challenging, especially linked to the frequently overlapping patterns among subtypes of these tumors [27].

#### 2.2.1. Salivary Duct Carcinoma (SDC)

**Figure 2:**
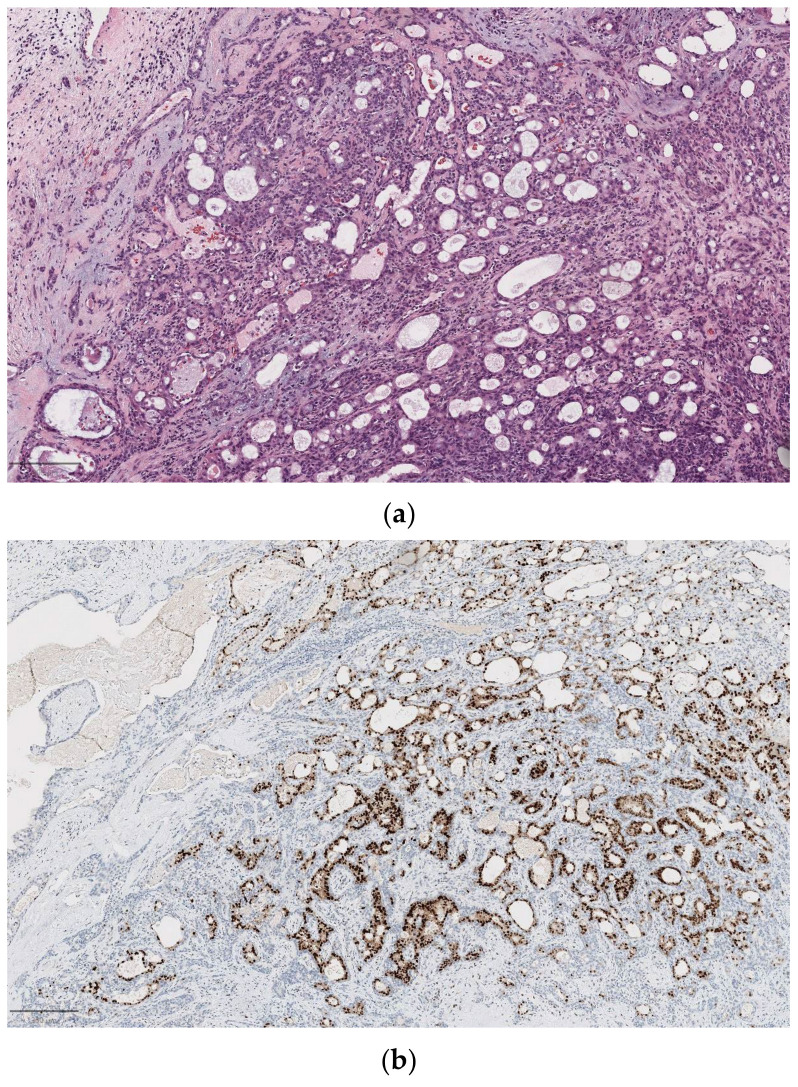

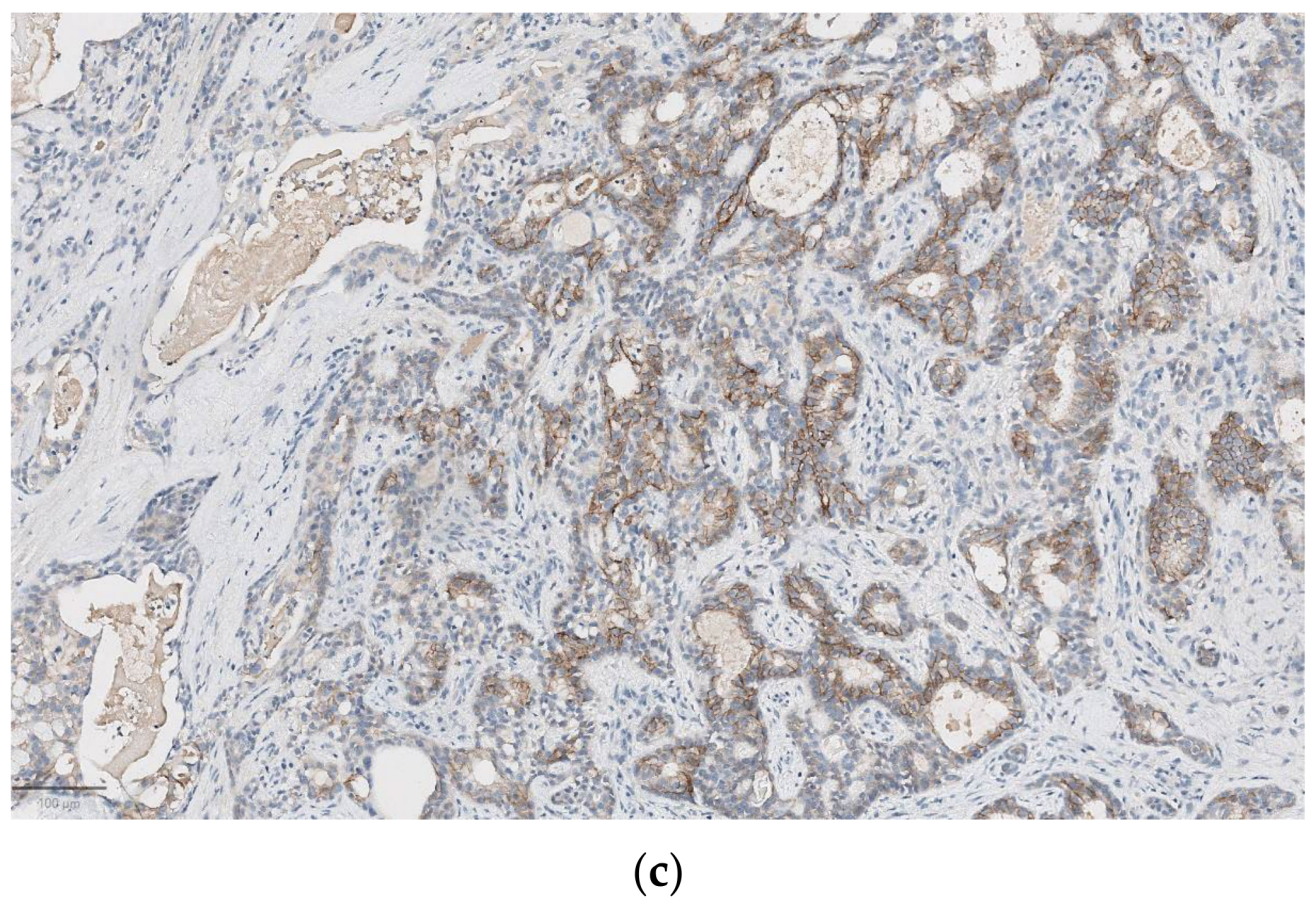
Salivary duct carcinoma in pleomorphic adenoma (carcinoma ex pleomorphic adenoma). (**a**) On H&E (5×), complex architecture with cribriforming glands and a Roman-bridge pattern; (**b**) strong immunopositivity for androgen receptors (ARs) in salivary duct carcinoma on the right and negativity of the component of pleomorphic adenoma; (**c**) HER2 overexpression cells: a strong complete membrane staining is observed in >10% of tumor cells.

Among SGCs, salivary duct carcinoma (SDC) is the entity with the worst prognosis, showing a 5-year overall survival (OS) of 40–65% and a 4-/5-year disease-free survival (DFS) of 17–44% [28]. SDC can present a wide range of immunohistochemical expressions [29]. Recent studies analyzing the molecular and histologic profiles of SDC indicate that the androgen receptor (AR) signaling pathway plays a role in tumor progression and aggressiveness [30,31] (Figure 2).

The immunoprofiles of these tumors suggest that clinical outcomes and treatment responses may be influenced by the receptor status of AR, HER-2/Neu, PIK3CA, and others. Additionally, some researchers suggest that an apocrine phenotype and AR expression are essential for SDC diagnosis, proposing that salivary malignancies not meeting these criteria could be reclassified as different cancer subtypes [32].

#### 2.2.2. Mucoepidermoid Carcinoma (MEC)

Concerning the major salivary glands, MEC is the most common histological subtype, with 89% of cases arising in the parotid gland. Histologically, MEC is characterized by a poorly circumscribed mass without a capsule [33].

**Figure 3:**
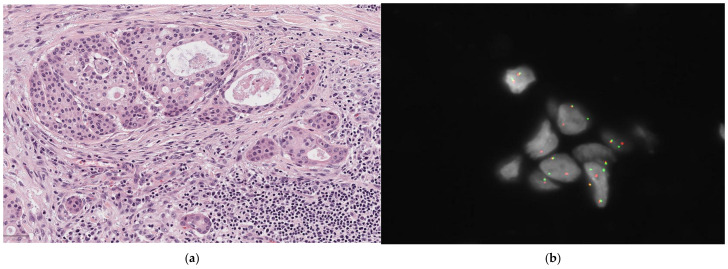
Mucoepidermoid carcinoma. (**a**) On H&E (10×), cystic spaces are partly lined by mucous cells. Other cell types include intermediate, squamoid, and partly clear cells; (**b**) FISH result positive for MAML2 rearrangement.

MEC is characterized by the presence of mucous (mucin-producing) cells, intermediate cells, and epidermoid (squamoid) cells. The tumor’s architecture includes both cystic and solid areas, with varying proportions of these cell types. The cystic spaces are partially lined by mucous cells, which have abundant mucinous cytoplasm and nuclei located on the periphery. These cells show intracytoplasmic PAS staining that is resistant to diastase. Extracellular mucin may also be found. Epidermoid (squamoid) and intermediate cells are often the most common type in these tumors. Significant keratinization should be considered an exclusion criterion for the diagnosis of MEC [21,33,34] (Figure 3).

Various grading systems have been proposed [35,36,37], none of which has reached consensus. According to Cipriani et al. [38], who compared the prognostic impact of the different grading systems, the one proposed by Brandwein [36] is the most reliable. Nevertheless, this statement has recently been criticized by Katabi et al. because Brandenwein’s grading also includes criteria usually applied for staging [39]. Nevertheless, features such as nuclear pleomorphism, mitotic figures, necrosis, and perineural and lymphovascular invasion are generally considered as high-grade features.

#### 2.2.3. Adenoid Cystic Carcinoma (ACC)

Adenoid cystic carcinoma (ACC) occurs most commonly in the submandibular gland and is the predominant histological subtype among SGCs of the nasopharynx (constituting approximately 43% of cases in the Caucasian population) [40]. ACC is composed of two primary cell types: ductal cells and myoepithelial cells. Ductal cells have an eosinophilic cytoplasm with uniform round nuclei, while myoepithelial cells feature a clear cytoplasm and hyperchromatic, angular nuclei. Mitotic figures are rare. A key characteristic of ACC is perineural invasion, which depends on the extent of sampling. ACC exhibits three distinct growth patterns: tubular, cribriform, and solid [21]. The tubular pattern involves well-formed ducts and tubules lined by luminal ductal cells and abluminal myoepithelial cells. The cribriform pattern, which is the most common, is characterized by nests of tumor cells with microcystic-like spaces filled with hyaline or basophilic mucoid material. These spaces are pseudocysts contiguous with the tumor stroma. The solid pattern is defined by sheets of basaloid cells lacking tubular or cribriform formations. It is common to see combinations of these growth patterns. Generally, ACCs with more than 30% solid components are associated with a more aggressive clinical course [21].

For diagnosis, identifying both pseudocysts and true glandular lumina is usually necessary. ACC with high-grade transformation is marked by a pleomorphic, mitotically active high-grade carcinoma component; the high-grade component usually does not express myoepithelial markers [41].

Differentiating the three ACC variants (classic, solid, and high-grade transformation) has a great prognostic impact [42]. The high-grade areas display a solid and sheet-like growth pattern, accompanied by necrosis, an elevated mitotic rate, and nuclear pleomorphism. The loss of the biphasic pattern, which is more clearly revealed through IHC, aids in recognizing high-grade transformation. In summary, the typical IHC features for high-grade transformation include the following: loss of the biphasic pattern, retention of AE1/AE3 and c-KIT expression, and reduced or focal expression of myoepithelial markers. Proliferation markers show Ki-67 at 30% (ranging from 50% to 70%) and p53 at 25% (ranging from 5% to 100%). Additionally, in molecular analysis, MYB/NFIB fusion and chromosomal gains are commonly observed. In contrast, conventional ACC exhibits a biphasic epithelial/myoepithelial pattern. The Ki-67 proliferation index ranges from 5% to 30%, while p53 expression is typically low, around 5% (0–5%). Additionally, it is characterized by MYB/NFIB chromosomal loss [42].

#### 2.2.4. Other Rare SGCs

Thoroughly documented new entities have been included in the last WHO edition as microsecretory adenocarcinoma (MSA) and sclerosing microcystic adenocarcinoma [21].

MSA is a low-grade carcinoma with an intercalated duct-like phenotype, typically showing MEF2C::SS18 fusion [7]. Histologically, it is characterized by microtubules and microcysts containing basophilic secretion and lined by small cells having an eosinophilic or clear cytoplasm. The cells typically lack marked atypia; no necrosis or intense mitotic activity is seen. MSA tends to infiltrate locally and lacks a capsule. MSA, if radically excised, has indolent behavior [21,43]; recurrences and distant metastases are rare events [7].

Cases of sclerosing microcystic adenocarcinoma have been reported in the oral cavity and other mucosal head and neck sites [44]. It is composed of cords and nests of neoplastic cells immersed in a desmoplastic stroma, exhibiting a biphasic cellular pattern that includes peripheral myoepithelial cells and cuboidal ductal cells, with eosinophilic secretions within the ducts. This tumor subtype is characterized by the positivity of luminal cells in immunohistochemistry for cytokeratin 7, while the peripheral myoepithelial cells show positivity for smooth muscle actin, S100, p63, and p40 [21].

In the salivary glands, adamantinoma-like Ewing sarcoma (ALES) is a rare variant of Ewing sarcoma characterized by complex epithelial differentiation, including the expression of cytokeratin and p40, and often exhibiting keratin pearl formation. ALES shares features with a wide range of tumors with basaloid or myoepithelial characteristics. Notably, a subset of high-grade myoepithelial carcinomas of salivary origin may also have EWSR1 rearrangements and show nuclear uniformity, a clear cytoplasm, a myxoid to hyalinized matrix, and p40 positivity, which can resemble ALES [45].

#### 2.2.5. Molecular Targets in SGCs

Up to now, molecular drivers have had a prevalent diagnostic role rather than a prognostic and therapeutic one.

Several studies have identified specific chromosomal rearrangements, such as CRTC1-MAML2 and CRTC3-MAML2, which have a diagnostic value in both low- and high-grade MECs. These two rearrangements are also associated with improved survival rates [46] [Figure 3b].

The fusions of MYB-NFIB and MYBL1-NFIB genes are prevalent in most ACCs of salivary glands [21], while SDC exhibits several chromosomal alterations, including human epidermal growth factor receptor 2 (HER2) amplification, PIK3CA, p53 or HRAS mutations, PTEN expression loss, and RET-NCOA4 fusion [47]. Moreover, a recent target of interest in ACC is the prostate-specific membrane antigen (PSMA), which is commonly expressed in several SGC subtypes; preliminary findings with 177Lu-PSMA radioligand therapy in ACC and SDC showed limited results with only stable disease achieved in ACC and only early progression in SDC [48].

Concerning new histotypes, a specific MEFC2-SS18 fusion has been identified exclusively in MSA [43].

Further molecular alterations, such as abnormalities in PLAG1 and HMGA2 genes, AKT1 E17K mutations, or PRKD1-3 gene translocations, have been observed in pleomorphic adenocarcinoma, mucinous adenocarcinoma, and cribriform adenocarcinoma of minor salivary glands, respectively [46].

In SDC and adenocarcinoma NOS, frequently characterized by HER2 gene overexpression, followed by MEC, HER-2-targeted therapies represent a potentially effective therapeutic option [49]. This is demonstrated by some case reports showing a complete response obtained with trastuzumab-based therapies in SDC.

Notably, studies on HER-2-positive SGCs employ treatment regimens similar to those used for breast cancer, such as HER-2 antibodies (trastuzumab, pertuzumab, and TDM-1) in combination with taxane-based chemotherapy. According to some studies, though involving a small number of patients, the overall response rate (ORR) varies between 67% and 100%, with an mPFS ranging from 3 to 18 months [50,51].

More recently, the results observed with trastuzumab deruxtecan in R/M HER2-positive SDC were promising in terms of efficacy and tolerability [52].

Since SGCs are microsatellite-stable and present a low tumor mutational burden (TMB), the potential of immunotherapy appears limited, although further research is warranted [8].

Another therapeutic approach involves targeting androgen receptor (AR) expression, present in approximately 64–98% of SDC. Specifically, in a retrospective analysis of patients with recurrent/metastatic AR-positive SGCs treated with androgen deprivation therapy (ADT), the ORR was around 65% [53]. However, the final results of EORTC1206, recently presented at ESMO, showed that ADT (a combination of triptorelin and bicalutamide) did not outperform chemotherapy as a first-line treatment for AR-expressing SGCs patients. On rare occasions, SGCs present mutations in NTRK or RET genes, for which TKIs (i.e., entrectinib for NTRK fusion or selpercatinib for RET rearrangements) may be promising treatments [54].

### 2.3. Sinonasal Carcinoma

Sinonasal tumors account for less than 1% of all cancers and about 3% of HNCs, with SCC being the most prevalent histological subtype [55]. These tumors are most commonly found in the nasal cavity, followed by the maxillary sinus and, less frequently, the frontal sinus [56].

The prognosis for sinonasal tumors is often poor due to the difficulties in diagnosing these malignancies at early stages, which are caused by their complex anatomical location and the wide variety of histological subtypes [57].

The complexity of histopathological entities in this area is such that diagnosis is based on the immunohistochemical and molecular exclusion of overlapping entities [58].

Regarding histology, sinonasal SCC can be both keratinizing and non-keratinizing; other histological subtypes that are typically found elsewhere in head and neck district (e.g., verrucous, basaloid, spindle cell carcinoma) are rare. Recent studies have found a higher incidence of transcriptionally active HPV, including type 16, 31/33, and 18, in some variants of sinonasal SCC, such as basaloid (46%), papillary (80%), and adenosquamous (67%) [59,60].

Sinonasal keratinizing SCC has similar morphological features to conventional SCC arising from other head and neck sites, with irregular nests and cords of eosinophilic cells demonstrating keratinization and inducing a desmoplastic stromal reaction.

Non-keratinizing SCC is characterized by cylindrical non-keratinizing cells and a sharp demarcation at the epithelium–stroma interface (ribbon- or garland-like patterns) with central necrotic areas and tumor cells arranged perpendicularly to the tumor–stroma interface. An etiologic relation with transcriptionally active high-risk HPV [61] has been suggested, which may explain the more favorable outcome [62].

Sinonasal undifferentiated carcinoma (SNUC) is a malignant epithelial tumor without any identifiable line of differentiation (including squamous, glandular, and neuroendocrine) and is a diagnosis of exclusion. If EBV or HPV is detected, the diagnosis of SNUC should be questioned. SNUC is composed of cells with medium- to large-sized nuclei, with single or multiple nucleoli, and arranged in nests, lobules, trabeculae, or sheets. In immunohistochemistry, the tumor is positive for pan-cytokeratins and simple keratins but is negative for CK5/6, which differentiates them from poorly differentiated small-cell sinonasal tumors [63,64,65].

SNUC is a high-grade epithelial neoplasm characterized by a lack of cell differentiation. Its diagnosis is made by excluding other sinonasal and non-sinonasal malignancies. Historically, many undifferentiated epithelial neoplasms were classified under SNUC until advancements in molecular pathology allowed for their reclassification into distinct entities. SNUC is an aggressive disease with a poor prognosis. Recently, mutations in isocitrate dehydrogenase 1 or 2 (IDH1/2) have been identified in a subset of SNUC cases [66,67].

The immunohistochemical detection of IDH1/2 mutations offers a more cost-effective alternative to molecular genetic testing, although it cannot capture the full range of these mutations. The standard treatment approach involves a combination of surgery, chemotherapy, and radiation. Tumors with IDH2 mutations tend to have a less aggressive clinical course compared to other SNUCs. IDH mutations also present potential therapeutic options, including selective small-molecule inhibitors like Enasidenib (for IDH2 mutations) and Ivosidenib (for IDH1 mutations) [68,69].

Moreover, a recently recognized entity known as HPV-related multiphenotypic sinonasal carcinoma (HMSC) has been introduced [69]. HMSC is an epithelial tumor almost exclusively localized in the sinonasal tract and is associated with high-risk HPV, most commonly type 33. P16 immunohistochemistry can be used to identify the HPV association.

HMSC histologically resembles adenoid cystic carcinoma but is also associated with features of in situ squamous cell carcinoma. Although it typically appears high-grade with destructive growth and a tendency for local recurrence, HMSC has a low potential for metastasis and rarely exhibits lethal behavior [70,71].

**Figure 4:**
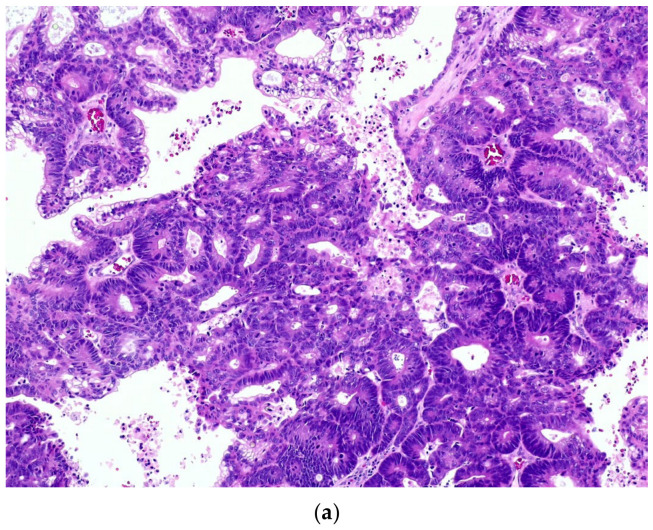

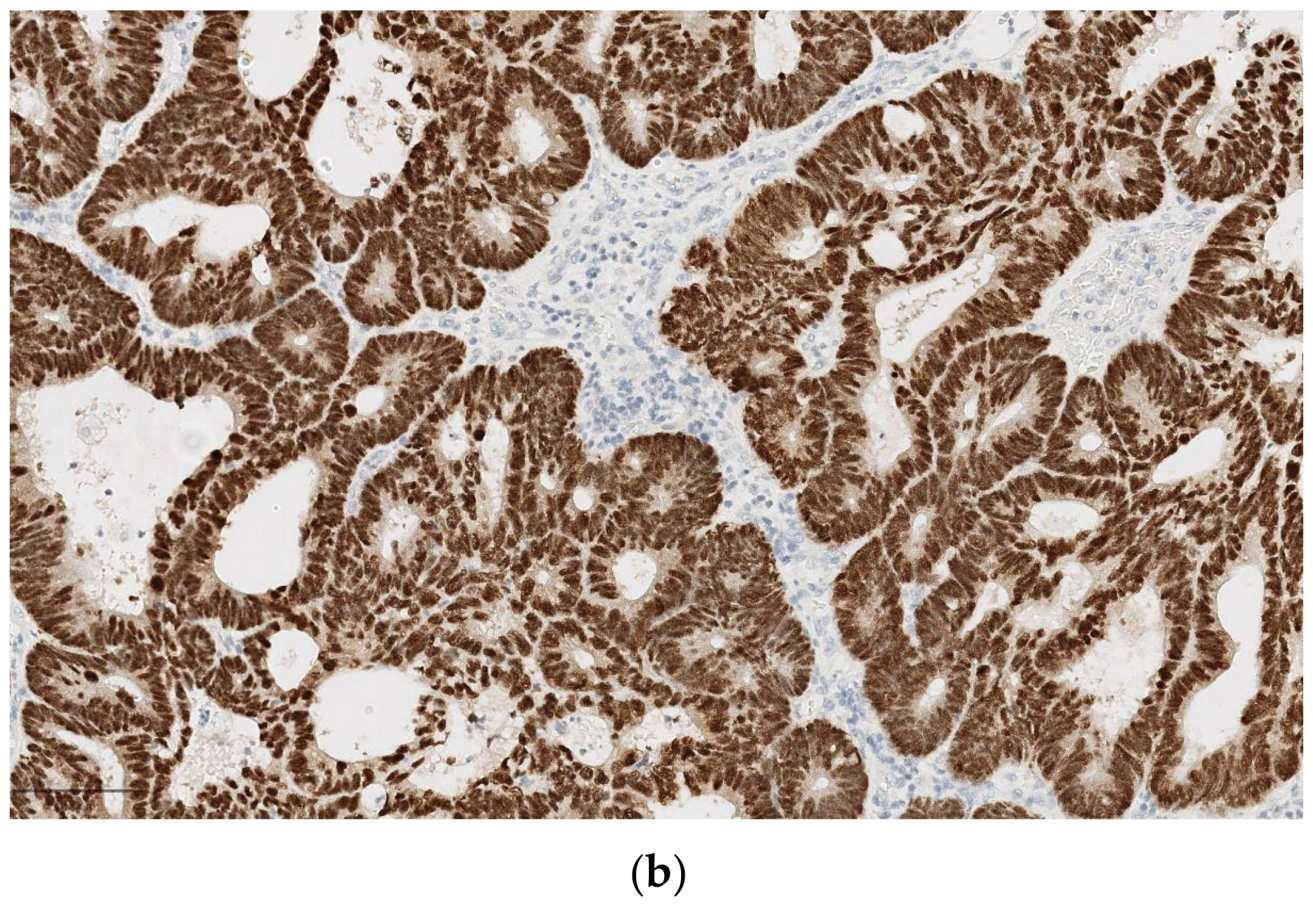
ITAC. (**a**) A moderately differentiated tumor with a tubular arrangement of neoplastic cells on H&E (10×); (**b**) the tumor cells show immunoreactivity for CDX2, supporting their intestinal differentiation.

In the sinonasal tract, three different types of adenocarcinomas may be present. While the salivary type, presumably originating from the seromucinous glands, tends to be similar to other salivary-type HNCs, intestinal-type adenocarcinoma (ITAC) and non-ITAC are unique entities of the sinonasal tract. ITACs are closely related to professional exposure to wood and/or leather dust, are morphologically similar to primary colorectal adenocarcinomas, and mostly involve the ethmoid sinus [72]. They consist of proliferations of columnar cells with interspersed goblet cells, forming papillae and glands. Paneth cells and endocrine cells are also present in varying proportions. Differentiation varies from very-well-differentiated to poorly differentiated tumors classified as papillary, colonic, solid, or mixed. Well-differentiated ITACs predominately show a papillary or tubulopapillary architecture and consist of elongated outgrowths lined by atypical intestinal-type cells. Sometimes atypia may be difficult to appreciate, but nuclear changes that appear at least adenomatous are the rule. The nuclei are cigar-shaped, hyperchromatic, and enlarged. As ITACs become poorly differentiated, tubular and papillary structures are replaced by nested, cribriform, and solid growths. Mitotic figures are frequent, and central comedonecrosis is usually present. Neoplastic cells have a peculiar immunohistochemical pattern (CK20, CDX2, and MUC2 and villin) and present KRAS mutations in 6–40% of cases, whereas BRAF mutations occur in <10% [70] (Figure 4).

Non-intestinal-type sinonasal adenocarcinoma (SNAC) is a sinonasal tract gland-forming malignancy lacking intestinal-type features and the characteristics of salivary gland-specific tumor entities.

Non-ITACs may be divided into low-grade, with various architectural patterns (glandular, tubular, papillary, cystic), and high-grade tumors, characterized by solid growth patterns, necrosis, and a high number of mitosis and pleomorphic cells [73]. The tumors express cytokeratins (typically CK7 and infrequently CK20) while usually lacking markers of intestinal differentiation (CDX2 and MUC2).

NUT carcinoma can occur in the nasal cavity and paranasal sinuses. It is a poorly differentiated carcinoma, often showing squamous differentiation and focal keratinization, and is defined by the presence of a nuclear protein in testis (NUT) gene rearrangement. Its rarity makes the exact incidence and etiology unclear. Clinically, NUT carcinoma in this location presents with symptoms such as nasal obstruction, pain, epistaxis, nasal discharge, and frequently eye-related issues like proptosis. In about 50% of cases, it is associated with lymph node involvement or distant metastasis [74]. Diagnosis is based on the detection of the NUT rearrangement rather than histological appearance. In addition to NUT, other common positive markers include p63, p40, and cytokeratins. CD34 is often expressed, and occasionally, neuroendocrine markers, p16, and TTF1 may also be present. Due to its poorly differentiated nature, NUT carcinoma can be misdiagnosed as poorly differentiated squamous cell carcinoma. The prognosis is generally poor, with a median overall survival of 9.8 months. Currently, there is no established standard treatment for HN-NUT carcinoma. In clinical practice, a multimodal approach combining systemic chemotherapy, surgery, and radiation therapy is typically used. Recently, two promising classes of targeted therapies, BET inhibitors and histone deacetylase inhibitors, have shown potential and are being explored in clinical trials for adults with NUT [75].

Previous studies have demonstrated high levels of PD-L1 expression both in ITAC (26%) and sinonasal SCC (30–46%) [76,77,78] and the presence of CD8+ tumor infiltrating lymphocytes (TILs), predominantly in the squamous type (up to 57%); even microsatellite instability (MSI) is reported in 21% of sinonasal SCC [79]. De Cecco et al. identified a strong association between SNUCs and immune-system-related functional pathways, suggesting that these tumors may be more responsive to immunotherapeutic strategies due to their increased potential to elicit an immune response [80].

These results indicate that a substantial subset of patients with poorly differentiated sinonasal tumors may be candidates to be treated with immunotherapy [81,82]. Moreover, a recent in vitro study demonstrated that targeting PD-L1 on SNUC cells using an anti-PD-L1 antibody not only significantly enhanced the immune response against tumor cells but also increased PD-L1 expression in cells that were previously PD-L1-negative, providing the rationale to use immunotherapy alone or in combination in this setting [83]. Notably, a case report showed a durable response with dual checkpoint inhibition (pembrolizumab and ipilimumab) in the metastatic setting, after progression to the first immune checkpoint inhibitor [84].

### 2.4. Mucosal Melanoma

Primary mucosal malignant melanomas (MMs) of the head and neck region account for 0.8-3.7% of all melanomas and 0.03% of all cancers [85].

MMs are a rare type of neural-crest-derived neoplasms that can arise from stem melanocytes and mature melanocytes of the submucosa that have acquired genetic alterations. MMs are biologically distinct from cutaneous melanomas. The etiological factors, including melanocytosis, remain unclear [86,87,88].

**Figure 5:**
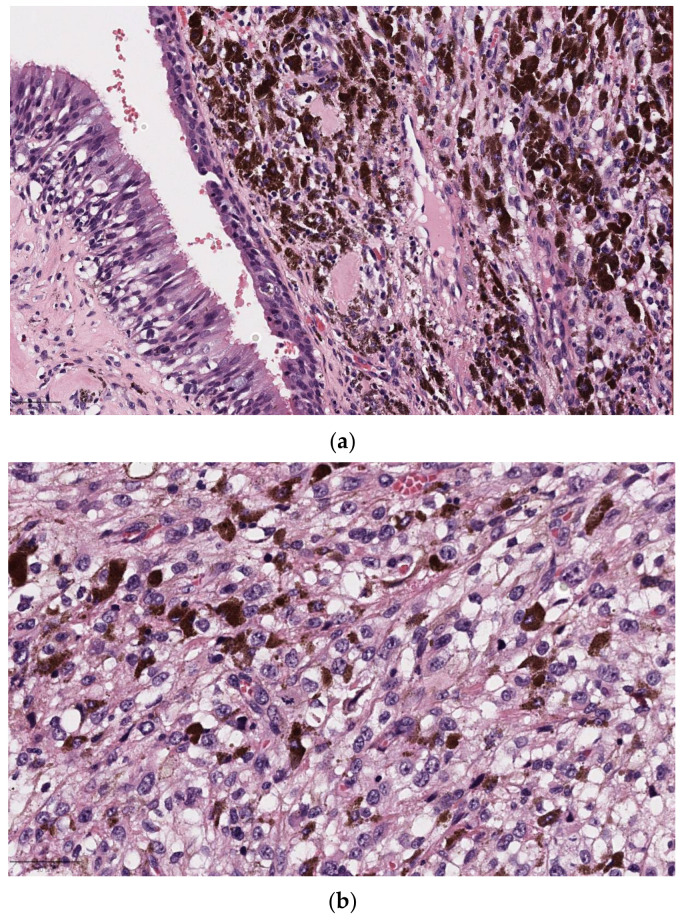
Malignant melanoma. (**a**) (10×) Epithelioid tumor cells infiltrating the basal layer of squamous epithelium as single cells on the left and the nodular infiltrating component on the right; (**b**) at higher magnification (40×), epithelioid tumor cells with amphophilic cytoplasm, large nuclei, and prominent nucleoli are arranged in solid sheets. Melanin is present.

The tumor cells display a wide range of morphologies, including spindled, epithelioid, plasmacytoid, rhabdoid, round, clear, and undifferentiated forms, often within the same tumor. Epithelioid tumor cells possess amphophilic hyaline cytoplasm and atypical nuclei with a high nucleus-to-cytoplasm (N/C) ratio and prominent macronucleoli, organized in solid sheet-like or nested growth patterns. Spindled tumor cells are arranged in intersecting fascicles. Invasive MMs predominantly affect the submucosa but can infiltrate the underlying bone and cartilage. Mitoses are commonly observed and often atypical. Necrosis and cellular discohesion frequently create a pseudopapillary or peritheliomatous pattern, with a pseudoalveolar pattern also being reported [89] (Figure 5). Surface ulceration is often present but with an intact surface epithelium; pagetoid and/or surface spread may be present.

Up to 50% of lesions are amelanotic, leading to a complex differential diagnosis [85,88,89].

S100 and melanocytic markers (HMB45, tyrosinase, melan-A, MITF, SOX10) are differently expressed depending on the morphological subtype. Recently, PRAME (Preferentially-expressed Antigen in MElanoma) has been reported to have diagnostic and some prognostic value in cutaneous melanomas and certain head and neck malignancies [90,91]. The molecular profile of MM significantly differs from that of cutaneous and uveal melanomas, showing a lower frequency of somatic mutations and fewer UV-related genetic alterations typically observed in cutaneous melanoma. MM is characterized by higher rates of KIT mutations/amplifications (25%), followed by NRAS mutations (15–20%) and rare BRAF mutations (<6%) [85,87].

For early-stage MM of the head and neck region, treatment involves radical surgery with clear margins, as this is a crucial prognostic factor. Postoperative RT is recommended due to its demonstrated ability to reduce local recurrence, although its effect on the OS remains uncertain [92]. Despite treatments, a significant number of patients still experience local recurrence [93].

Although MM exhibits biological differences compared to cutaneous melanoma, targeted therapies may represent a promising treatment option. However, scientific evidence is very limited, as metastatic mucosal melanoma is a rare entity. Among TKIs, there are slightly more data available on MM regarding Imatinib, given that a study identified a cKIT mutation in approximately 39% of MM [94]. A phase II study involving patients with MM harboring the cKIT mutation or amplification demonstrated durable responses with Imatinib in up to 16% of cases [95].

Regarding immunotherapy, which has revolutionized the treatment of cutaneous melanoma, its efficacy in MM remains uncertain due to very limited data, all of which were derived from retrospective analysis [91]. In KEYNOTE-001/002/006, a post hoc analysis of MM patients demonstrated a lower ORR and overall survival with pembrolizumab compared to cutaneous melanoma [96]. Also, the combination of immune checkpoint inhibitors (nivolumab and ipilimumab) has an unclear role in MM, probably with modest efficacy in this population [97]. Another option is represented by the combination of anti-VEGF antibodies and other systemic treatments (for example, bevacizumab with chemotherapy or with atezolizumab), which showed an improvement in the ORR and PFS [98,99].

### 2.5. Hematolymphoid Malignancies of Head and Neck

In the fifth edition of the WHO classification of HNCs, hematolymphoid malignancies were substantially reorganized and expanded, including also lymphoid proliferations, previously treated among other types of neoplasms of a specific anatomic site [100].

Primary head and neck hematolymphoid neoplasms are a heterogenous group of malignancies originating from B cells, plasma cells, T cells, NK cells, and histiocytic and dendritic cells and primarily erupting from the head and neck region. Lymphoid neoplasms account for approximately 5% of all types of HNCs and may develop with nodal or extranodal involvement [101].

Malignant lymphoma is traditionally classified into two subtypes: Hodgkin’s lymphoma (HL) and non-Hodgkin’s lymphoma (NHL), with the latter being the most common in the head and neck region (75%). In both subtypes, cervical lymphadenopathy is the most common presentation, with HL frequently involving lower cervical or supraclavicular nodes; mediastinal and hilar nodes are often affected as well.

Additionally, the head and neck region is the second most commonly involved anatomical location of extranodal lymphoid neoplasms [102], arising from Waldeyer’s ring (palatine and lingual tonsils—the most affected—nasopharyngeal adenoids and base of tongue) in half of cases [103]. Laryngeal lymphomas, including diffuse large B-cell lymphomas (DLBCLs) and MALT lymphomas, are usually located in the supraglottic region. Lymphoma of the salivary glands accounts for approximately 2–5% of salivary gland neoplasms, generally involving the parotid gland and mostly represented by marginal-zone B-cell lymphoma of the MALT type (from parotid parenchyma), follicular lymphomas (from intraparotid lymph nodes), and DLBCLs.

In Waldeyer’s ring, the most frequent histopathological forms are DLBCLs (50% of cases), follicular, Burkitt’s, and mantle cell lymphomas [104].

DLBCLs usually demonstrate a positive reaction with pan-B-cell markers, particularly for follicular center cell-derived cells (CD45RB, CD20, CD79a, bcl-6, CD10, vimentin, and p63), and they are non-reactive with T-cell markers. The proliferation index (Ki-67) is usually greater than 90%. The cells are negative with CD3, CD5, CD56, and EBV early RNA (EBER), differentiating them from NK/T-cell lymphoma. Reactive lymphoid follicular hyperplasia, infectious mononucleosis, and NK-NPC need to be excluded histologically and/or immunophenotypically [105].

Other peculiar head and neck malignancies are extranodal NK/T-cell lymphomas (ENKTLs), which are extranodal EBV-related malignancies, mostly of NK-cell and occasionally of T-cell lineage, divided into nasal lymphoma (involving the nasal and/or paranasal region) and extranasal lymphoma (arising from the skin, gastrointestinal tract, testis, or soft tissue).

**Figure 6:**
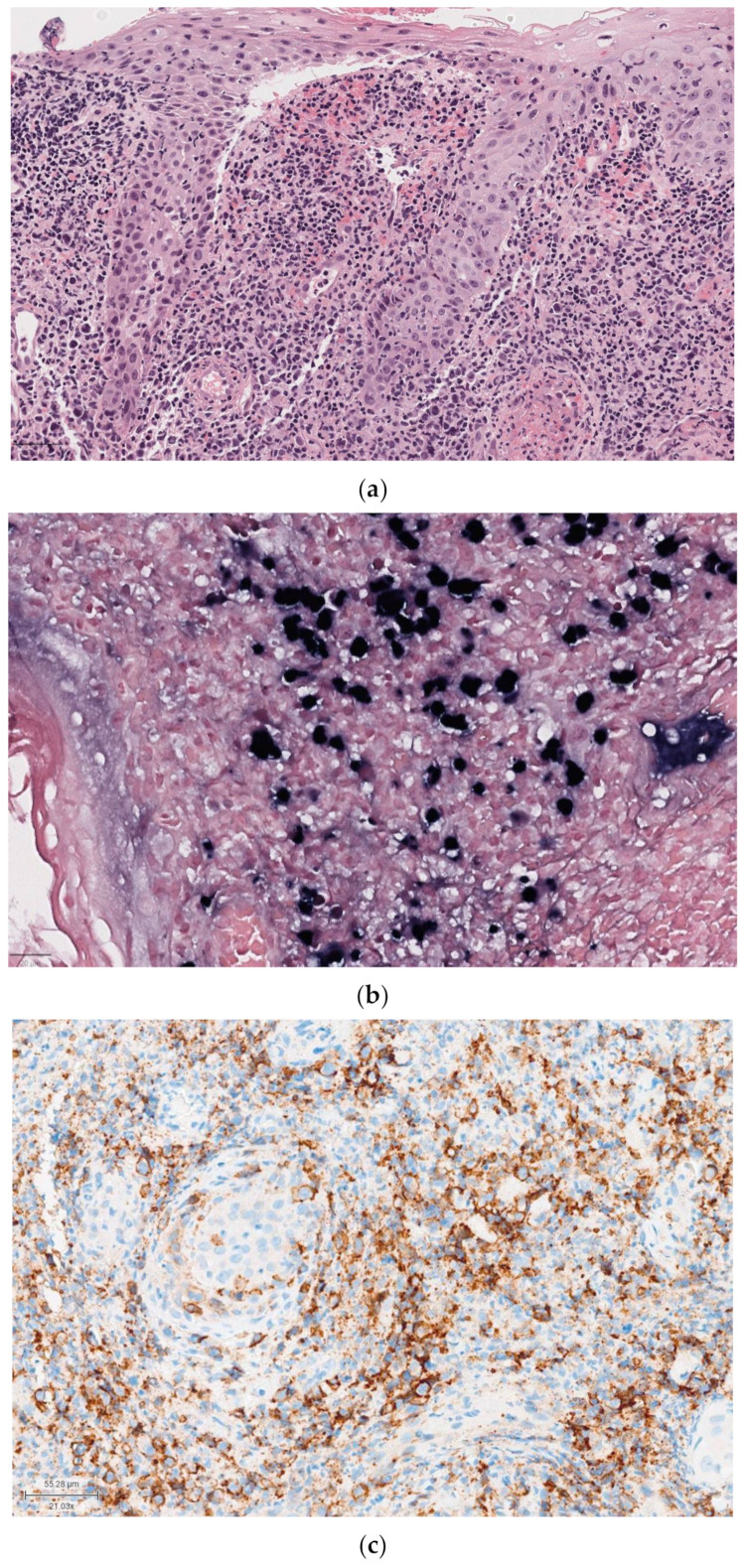
Extranodal NK/T-cell lymphoma (ENKTL). (**a**) (10×) The lymphoma shows medium to large tumor cells with irregular and often hyperchromatic nuclei associated with nested hyperplastic squamous epithelium; (**b**) positive labelling of the tumor cell nuclei for EBV-encoded small RNAs (EBERs) on in situ hybridization; (**c**) positive CD56 in neoplastic cells.

Pathologically, these lymphoma cells often show angiocentricity and angiodestruction, resulting in zonal necrosis. Extensive inflammation associated with a tumor makes diagnosis difficult because of the high risk of false negatives. ENKTL exhibits a polymorphous infiltration of neoplastic cells mixed with inflammatory cells; a florid pseudoepitheliomatous hyperplasia of the overlying squamous epithelium could be present [Figure 6a]. The cytomorphology of the tumor cells varies and is most often composed of medium-sized cells or a mixture of small and large lymphocytes with hyperchromatic and irregular nuclei. The typical immunophenotype of ENKTL is CD20^−^, sCD3^−^, cCD3^+^, CD4^−^, CD5^−^, CD8^−^, and CD56^+^, with the expression of cytotoxic molecules such as TIA1, granzyme B, and perforin [106]. Lymphoma cells are almost invariably infected by EBV, which exists in a clonal episomal form that is not integrated into the host genome [107]. EBV infection is typically identified through EBER, which is a prerequisite for diagnosing NK/T-cell lymphoma, but this alone is insufficient [Figure 6b]. Either CD56 or cytotoxic molecules must also be present for a definitive diagnosis [Figure 6c] [108].

Regarding molecular pathology, conventional karyotyping has identified the deletion of chromosome 6q (6q–) as a recurrent cytogenetic aberration in NK/T-cell lymphoma; gene expression profiling (GEP) has shown similarities between NK/T-cell lymphoma and peripheral T-cell lymphoma (PTCL) of the gamma/delta subtype. Additionally, exome sequencing has revealed activating mutations in STAT3 and STAT5B in a significant number of cases. Next-generation sequencing has identified mutations in the RNA helicase gene DDX3X in approximately 20% of cases, TP53 mutations in about 13%, and, more rarely, alterations in genes involved in epigenetic pathways—such as MLL, ASXL1, ARID1A, and EP300 [106].

### 2.6. Neuroendocrine Carcinomas (NECs)

NECs, poorly differentiated epithelial neuroendocrine neoplasms, are aggressive neuroendocrine neoplasms that can be subclassified as small-cell NEC and large-cell NEC.

A recent comprehensive review of head and neck NECs documented, among the different head and neck sites, the sinonasal tract as being the most common site of occurrence, followed by the larynx, oropharynx, nasopharynx, oral cavity, and major salivary glands [109,110,111].

Large-cell neuroendocrine carcinoma (LCNEC) shows nested, organoid, or trabecular growth with frequent peripheral palisading, rosette formation, and central comedonecrosis. It is composed of cells larger than the diameter of three lymphocytes with round to oval nuclei, vesicular chromatin, a single prominent nucleolus, and eosinophilic cytoplasm. The tumor cells have abundant eosinophilic cytoplasm. The nuclei have coarse to speckled chromatin, often with a single prominent nucleolus. The mitotic rate is > 10 mitoses/2 mm2 but is generally much higher. Rare cases of nasopharyngeal LCNEC are EBV-positive.

Small-cell neuroendocrine carcinoma (SCNEC) is characterized by sheet-like and nested growth, with occasional trabeculae and rosettes.

The tumor cells display hyperchromatic or stippled nuclear chromatin. Features such as nuclear molding, apoptosis, and increased mitotic activity are evident, with the mitotic count typically exceeding 10 mitoses per 2 mm^2^, often reaching even higher levels. The presence of crush artifacts and extravasated DNA coating blood vessels (known as the Azzopardi phenomenon) is also commonly observed [103].

In SCNEC, the neoplastic cells exhibit immunoreactivity for cytokeratin (AE1/AE3 and CAM5.2 cocktail) and are nearly uniformly positive for chromogranin-A and/or synaptophysin. If chromogranin-A is absent, a diffuse expression of INSM1 is necessary to confirm the diagnosis [111]. Additionally, p16 is frequently overexpressed in SCNEC, irrespective of HPV status [112].

In the head and neck region, sinonasal carcinomas deficient in the SWI/SNF complex (e.g., SMARCA4- or SMARCB1-deficient sinonasal undifferentiated carcinomas) may express neuroendocrine markers and can resemble neuroendocrine carcinoma. It is crucial to note that most NECs typically have a very high Ki-67 proliferation index, often exceeding 55%, which helps distinguish them from other neuroendocrine neoplasms [111,113].

Besides the Ki-67 labeling index, NECs can be distinguished from NENs based on aberrant p53 and RB expression patterns: the loss of nuclear RB expression and p53 overexpression are characteristic features of NECs [114]. Somatostatin receptor (SSTR) immunohistochemistry can aid in this distinction, as most NENs tend to express SSTRs, unlike NECs [115].

### 2.7. Head and Neck Sarcomas and Other Rare Tumors

The majority of HNC sarcomas originate from soft tissue, and only 20% are bone or cartilage sarcomas. Head and neck soft tissue sarcomas (HNSTSs) comprise approximately 1% of HNCs and 10% of all soft tissue sarcomas [116,117]. They are associated with a higher risk of locoregional recurrence and mortality, along with significant morbidity, mostly due to the difficulty of accomplishing a radical surgical excision with wide margins in this anatomic district and to the inability to deliver adequate RT usually due to the proximity to critical structures [118]. Among more than 50 histopathological subtypes of sarcomas, in the head and neck district, the most frequent forms are pleomorphic sarcoma (or malignant fibrous histiocytoma), fibrosarcoma, angiosarcoma, malignant peripheral nerve sheath tumor, and non-classified/non-differentiated sarcoma [119,120].

The histological subtype in head and neck sarcomas is particularly relevant since there are major differences in the clinical characteristics, prognosis, risk classification, and irradiation dose required. For example, in head and neck rhabdomyosarcomas, the alveolar type, which is more common in patients with a previous primary HNC, is associated with PAX3-FOXO1 or PAX7-FOXO1 fusion, while the embryonal type, which has a more favorable prognosis, is characterized by the activation of the IGF2 pathway [121]. Molecular analysis may be useful in the case of histological types with a low response rate to conventional chemotherapy, such as osteosarcomas: several in vitro and preclinical studies showed the expression of platelet-derived growth factor receptors (PDGFRs), VEGFRs, and RET in osteosarcomas; thus, multi-kinase inhibitors can be evaluated as future potential therapeutic tools [122,123].

**Figure 7:**
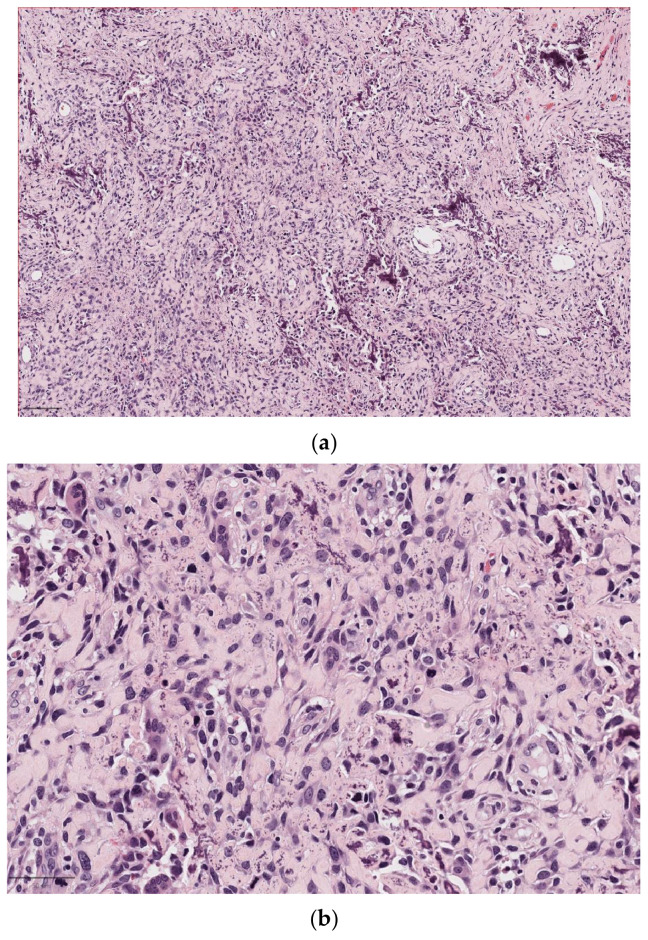
High-grade osteoblastic osteosarcoma. (**a**) Malignant-appearing polyhedral and spindle cells producing an immature osteoid matrix on H&E (10×); (**b**) at higher magnification (40×), neoplastic cells with a lace-like pattern. Abundant mitoses are revealed.

In addition to sporadic sarcomas, up to 0.3% of patients treated with radiotherapy for HNCs can develop a radiation-induced sarcoma [124,125], which commonly occurs 3 years or more after the RT [126] and is associated with a poor prognosis (Figure 7) [127]. In fact, radiation-induced sarcomas are characterized by a different gene profile expression from primary sarcomas, which results in low sensitivity to chemotherapy and radiotherapy, difficulty in potential surgical approaches, a higher local recurrence rate, and shorter disease-specific survival. Radiation-induced sarcomas seem to present higher immune cell infiltration and a higher microsatellite instability (MSI) rate than primary sarcomas; consequently, they may obtain better outcomes with a combination of immunotherapy and conventional chemotherapy (i.e., doxorubicin) [128].

Another rare entity in HNCs is represented by odontogenic tumors. The majority of odontogenic tumors are benign, while malignant ones account for about 6%, including carcinomas, sarcomas, or carcinosarcomas.

The odontogenic carcinosarcoma is an extremely rare histotype, firstly described by Tanaka et al., and presents with intermediate characteristics between odontogenic fibrosarcoma and ameloblastoma [129].

Ameloblastoma often has a benign behavior but potential aggressiveness, with its most frequent subtype being solid multicystic ameloblastoma (SMA). SMA is a slowly growing solid mass, predominantly in the tongue, oral cavity, and posterior mandible. From a molecular perspective, it is characterized by mutations in the MAPK pathway, such as BRAF V600E (present in almost 60% of mandibular SMA cases), FGFR2, or mutations in the RAS genes (HRAS, NRAS, KRAS) [129].

Ameloblastic carcinoma also tends to develop in the mandible, with a faster growth pattern, often leading to symptoms such as pain and paresthesia. Frequently, these tumors show alterations in the SOX2 gene, which helps distinguish them from ameloblastomas.

Clear cell odontogenic carcinomas are named for their histological presentation of Acid–Schiff-positive clear cells, polygonal in shape and embedded in fibrous stroma. These tumors often demonstrate positivity for p53 and high-molecular-weight cytokeratins [130].

**Figure 8:**
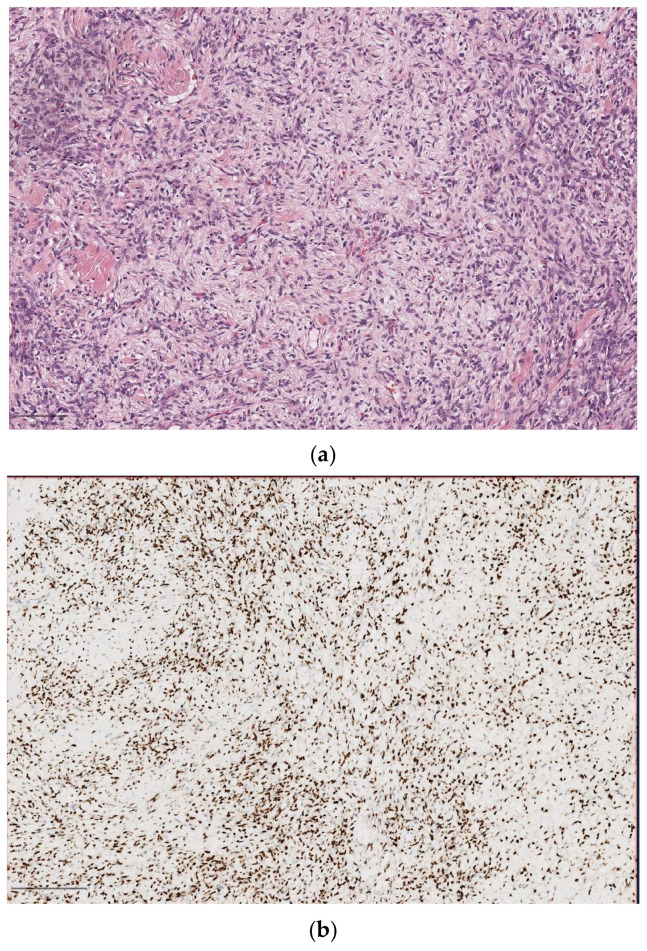
Solitary fibrous tumor. (**a**) On H&E (10×), refractile collagen associated with a modestly cellular, monotonous spindled cell neoplastic proliferation. The neoplastic cells have a syncytial quality, with bland, elongated, oval nuclei with scant cytoplasm; (**b**) a strong and diffuse nuclear reaction for STAT6 immunohistochemistry.

A solitary fibrous tumor (SFT) is an indolent tumor, with rare occurrence of metastasis. The most common sites involved by SFT in the head and neck are the sinonasal tract, orbit, oral cavity, and neck (Figure 8). The differential diagnosis is complex due to the histological features’ overlap; in fact, SFT can mimic several benign and malignant soft tissue tumors. SFT presents a mesenchymal phenotype, overexpressing the CD34 antigen and STAT6 (usually detected by immunohistochemistry), and is characterized by plump spindle cells in a collagenous background, low cell atypia, and low mitotic activity [131,132].

## 3. Conclusions

We provide an updated and detailed overview of the diagnostic and therapeutic challenges of r-HNCs, offering practical value to clinicians and researchers who manage these rare histological subtypes.

R-HNCs are characterized by a remarkably heterogeneous histological landscape and remain a diagnostic and clinical challenge. A correct pathological diagnosis, conducted by skilled head and neck pathologists, and molecular analysis, along with discussion within a multidisciplinary tumor board, are crucial to better handle HNC patients, particularly if affected by rare histological neoplasms, and lead to the appropriate therapeutic strategy.

## Figures and Tables

**Table 1:** Principal features of rare histopathological entities of the head and neck district. IHC: immunohistochemistry. NPC: nasopharyngeal carcinoma. SCC: squamous cell carcinoma. EBV: Epstein–Barr Virus. SGC: salivary gland cancer. SDC: salivary duct carcinoma. AR: androgen receptor. MEC: mucoepidermoid carcinoma. ACC: adenoid cystic carcinoma. SNUC: sinonasal undifferentiated carcinoma. HPV: Human Papillomavirus. ITAC: intestinal-type adenocarcinoma. SNAC: non–intestinal-type sinonasal adenocarcinoma. NEC: neuroendocrine carcinoma. DLBCL: diffuse large B-cell lymphoma. ENKTL: extranodal NK/T-cell lymphoma. NA: not assessed. MSI: microsatellite instability.

Tumor Type	Incidence * (×100.000/year)	Subtype	Histopathological Features	IHC/ Molecular Features	Prognostic Impact of Histological Type
NPC	0.11	Non-keratinizing SCC	Variety of architectural patterns (solid sheets, irregular islands, trabeculae, and isolated discohesive cells); varying numbers of lymphocytes and plasma cells.	EBV-positive.	Unclear
		Keratinizing SCC		EBV-positive (mostly in non-endemic areas).	
		Basaloid SCC		EBV-positive (mostly in non-endemic areas).	
SGC	0.96	SDC	Complex architecture with cribriforming glands and a Roman-bridge pattern.	Expression of AR, HER-2 expression/amplification, PIK3CA, p53, and HRAS mutations, PTEN expression loss, and RET-NCOA4 fusion.	Poor prognosis
		MEC	Cystic and solid areas. Presence of mucin-producing cells, intermediate cells, and squamoid cells in different proportions with intracytoplasmic PAS staining. No significant keratinization.	MAML2 rearrangement; HER2 expression/amplification.	Poor prognosis if high-grade features are present; better prognosis if MAML-2 rearrangement is detected
		ACC	Ductal cells and myoepithelial cells; presence of perineural invasion. Mixture of different growth patterns (tubular, cribriform, solid).	MYB-NFIB and MYBL1-NFIB gene fusions; PSMA expression.	More aggressive if high-grade transformation; poor prognosis if solid pattern is prevalent
Sinonasal carcinoma	0.45	Non-keratinizing SCC	Ribbon- or garland-like patterns; central necrotic areas and tumor cells arranged perpendicularly to the tumor–stroma interface.	HPV-positive in variants (basaloid, papillary, adenosquamous). MSI and PD-L1 positivity can be present.	Better prognosis
		Keratinizing SCC	Irregular nests and cords of keratinizing cells with desmoplastic stromal reaction.		Unclear
		SNUC	High-grade, undifferentiated cells; diagnosis of exclusion.	HPV- and EBV-negative. IDH1/2 mutations.	Poor prognosis
		ITAC	Columnar cells with interspersed goblet cells, forming papillae and glands. Paneth cells and endocrine cells present.	IHC-positive for CK20, CDX2, MUC2, and villin; KRAS and BRAF mutations. TP53 mutations.	Poor prognosis (grade 3)
		SNAC	Various architectural patterns, divided into low and high grade.	CK7 expression; lack of CDX2 and MUC2 expression.	NA
		NUT	Undifferentiated carcinoma or poorly differentiated squamous cell carcinoma; sheets of cells with moderately large, round to oval nuclei. Abrupt foci of keratinization.	NUT gene rearrangement; p63, p40, and cytokeratin positivity; CD34 is often expressed, and occasionally, neuroendocrine markers, p16, and TTF1 may also be present.	Poor prognosis
Mucosal melanoma	0.15		Epithelioid tumor cells: amphophilic hyaline cytoplasm and atypical nuclei with a high nucleus-to-cytoplasm (N/C) ratio, with solid sheet-like or nested growth patterns. Spindled tumor cells: intersecting fascicles.	HMB45, tyrosinase, melan-A, MITF, and SOX10 differently expressed. KIT mutations/amplifications; NRAS and BRAF mutations.	NA
NEC	NA	Large-cell NEC	Nested, organoid, or trabecular growth with peripheral palisading, rosette formation, and central comedonecrosis.	Rare EBV positivity.	Poor prognosis
		Small-cell NEC	Tumor cells are smaller than the diameter of 3 lymphocytes. Sheet-like and nested growth, with occasional trabeculae and rosettes. Azzopardi phenomenon is common.	AE1/AE3 and CAM5.2 expression, chromogranin-A and/or synaptophysin positivity. p16 positivity.	Poor prognosis
Head and neck sarcoma	0.26	Rhabdomyosarcomas	Alveolar type.	PAX3-FOXO1 or PAX7-FOXO1 fusion.	Poor prognosis
			Embryonal type.	IGF2 pathway alterations.	Better prognosis
		Osteosarcomas	Highly atypical cells producing neoplastic osteoid.	PDGFRs, VEGFRs, and RET mutations.	Poor response to conventional chemotherapy
		Radiation-induced sarcomas	Higher immune cell infiltration than sporadic sarcomas.	Higher immune cell infiltrations and MSI rate than sporadic sarcomas.	Poor prognosis
Ameloblastoma			Common types include follicular type and plexiform type. Others: acanthomatous, granular, basaloid, and desmoplastic.	BRAF, FGFR2 or RAS gene mutations.	Benign behavior
Ameloblastic carcinoma			Loss of the organized stratification of basal cells, stratum intermedium, and stellate reticulum.	SOX2 gene alterations.	Variable prognosis
Clear cell odontogenic carcinoma			Acid–Schiff-positive clear cells, polygonal in shape and embedded in fibrous stroma.	p53 and high-molecular-weight cytokeratin positivity.	Fair prognosis
Solitary fibrous tumor			Plump spindle cells in a collagenous background, low cell atypia, and low mitotic activity.	CD34 antigen and STAT6 expression.	Indolent tumor
Primary head and neck hematological malignancies		DLBCL		Positive reaction with pan-B-cell markers, particularly for follicular center cell-derived cells (CD45RB, CD20, CD79a, bcl-6, CD10, vimentin, and p63).	Poor prognosis
		ENKTL	Zonal necrosis. Polymorphous infiltration of neoplastic cells mixed with inflammatory cells; pseudoepitheliomatous hyperplasia of the overlying squamous epithelium.	CD20-, sCD3-, CD4-, CD5-, and CD8-negative; CD56- and cCD3-positive. TIA1, granzyme B, and perforin expression. EBV-positive.	Poor prognosis

* Incidences of different tumor subtypes are extrapolated from RARECARE.net.

## Data Availability

No new data were created or analyzed in this study. Data sharing is not applicable to this article.
